# Identification of DNA–protein Binding Sites through Multi-Scale Local Average Blocks on Sequence Information

**DOI:** 10.3390/molecules22122079

**Published:** 2017-11-28

**Authors:** Cong Shen, Yijie Ding, Jijun Tang, Jian Song, Fei Guo

**Affiliations:** 1 School of Computer Science and Technology, Tianjin University, Tianjin 300350, China; congshen@tju.edu.cn (C.S.); wuxi_dyj@tju.edu.cn (Y.D.); 2 Tianjin University Institute of Computational Biology, Tianjin University, Tianjin 300350, China; 3 School of Chemical Engineering and Technology, Tianjin University, Tianjin 300350, China; acthior@outlook.com; 4 Department of Computer Science and Engineering, University of South Carolina, Columbia, SC 29208, USA

**Keywords:** DNA–protein binding sites, ensemble classifier, feature extraction, random sub-sampling, sparse representation model

## Abstract

DNA–protein interactions appear as pivotal roles in diverse biological procedures and are paramount for cell metabolism, while identifying them with computational means is a kind of prudent scenario in depleting in vitro and in vivo experimental charging. A variety of state-of-the-art investigations have been elucidated to improve the accuracy of the DNA–protein binding sites prediction. Nevertheless, structure-based approaches are limited under the condition without 3D information, and the predictive validity is still refinable. In this essay, we address a kind of competitive method called Multi-scale Local Average Blocks (MLAB) algorithm to solve this issue. Different from structure-based routes, MLAB exploits a strategy that not only extracts local evolutionary information from primary sequences, but also using predicts solvent accessibility. Moreover, the construction about predictors of DNA–protein binding sites wields an ensemble weighted sparse representation model with random under-sampling. To evaluate the performance of MLAB, we conduct comprehensive experiments of DNA–protein binding sites prediction. MLAB gives MCC of 0.392, 0.315, 0.439 and 0.245 on PDNA-543, PDNA-41, PDNA-316 and PDNA-52 datasets, respectively. It shows that MLAB gains advantages by comparing with other outstanding methods. MCC for our method is increased by at least 0.053, 0.015 and 0.064 on PDNA-543, PDNA-41 and PDNA-316 datasets, respectively.

## 1. Introduction

DNA–protein interactions exert a crucial influence on diverse biological processes and is primal for cell metabolism. Contemporary researchers have scrutinized a considerable number of DNA and protein sequences including DNA-binding proteins. In addition, there is no lack of time-consumption in silico methods. Furthermore, the experimental determination of binding sites is always difficult and is not readily feasible all the time. Therefore, forecasting by statistical learning, which had been riveted by a lot of academics conducting surveys on DNA–protein binding sites, established in the field of computational and molecular biology, should be taken for granted. Several computational methods, which had been developed to identify DNA-binding sites in proteins, were generally based on protein sequence, protein structure or through integrating the aforementioned information. Most of these investigations are the methods that depended on machine learning techniques.

The concomitant information of sequence-based tactics [1,2] usually comprises physical and chemical properties of amino acids, evolutionary and other sequence information, such as BindN [3], BindN Random Forest (BindN-RF) [4], BindN+ [5], DNABindR [6], DNA Binding Sites Prediction (DBS-PRED) [7], DNA Binding Sites based on Position Specific Scoring Matrix (DBS-PSSM) [8], ProteDNA [9], DNA Protein-Binding (DP-Bind) [10], DNA Interaction Sites Identified from Sequence (DISIS) [11], Meta DNA Binding Site (MetaDBSite) [12], TargetDNA [13], etc.

Concretely, Wang et al. [3] take an amino acid sequence as input and extrapolate potential DNA or RNA-binding residues with Support Vector Machine (SVM). While the SVM model is constructed with encoded instances, which come from features w.r.t. three sequences. Specifically, the features are including side chain pKa value, hydrophobicity index and molecular mass of an amino acid. Wang et al. engage in knitting another craft [4] that feed the above information plus evolutionary information into Random Forest (RF) to realize Machine Learning (ML). We need to make another small point that the evolutionary information is represented by Position Specific Scoring Matrix (PSSM). Yan et al. [6] used Relative Solvent Accessible Surface area (RASA), sequence entropy, electrostatic potential and hydrophobicity as an input of Naive Bayes classifier (NB) to forecast binding sites. Ahmad et al. [8] create an Artificial Neural Networks (ANNs)-based algorithm and apply PSSM of amino acid sequences to predict DNA-binding sites. Cui et al. [9] handcraft a sequence based predictor, which was named ProteDNA, in order to taxonomize the residues in a Transcription Factor (TF) that implicates sequence-specific binding with DNA. The category of input feature originating from PSSM also contains a method that comes from Hwang et al. [10]. They conceive three kinds of ML methods including SVM, kernel logistic regression and penalized logistic regression to implement the prediction about binding sites. Ofran et al. [11] combine physicochemical features of sequence, PSSM, predicted secondary structure and Predicted Solvent Accessibility (PSA) to train a SVM model for predicting binding sites. Si et al. [12] consolidate the prediction results from six available online web servers: DISIS [11], DNABindR [6], BindN [3], BindN-RF [4], DP-Bind [10] and DBS-PRED [7], which only employ sequence information of proteins. Hu et al. [13] deploy PSSM and PSA to build a multiple SVMs model with weighted features. Georgiou et al. [14] use metric spaces and fuzzy sets to study entropy/clarity of genetic sequences. Buenrostro et al. [15] raise ATAC-seq to identify regions of open chromatin.

Structure-based knacks as a kind of classical methods usually apply structural motifs [16], secondary structure [17,18], Accessible Surface Area (ASA) [19] and Depth Index (DPX) [20] in DNA-binding residues identification. While several other kinds of methods collocate sequences with structure information to refine the performance of prediction [21]. Components like PSSM, ASA and Protein Backbone Structure(PBS) [22,23] are salient for the erection of forecasting model in these studies [19,24]. SVM [25], RF [26], ANNs or Bayesian Network (BN), clustering, network feature, PCVM [27] and Deep Learning [28] also play an imperative role in constructing the prognostic paradigm about DNA-binding residues identification [29,30] and speculation of other kind of bioinformatics data classification such as drug target interactions [31,32], protein–protein interactions [33,34], RNA-disease association [35], protein modification sites [36], DNA motif elucidation [37] and other related themes in computational biology [38,39,40].

No matter whether they are structure-based or methods of hybrid category, their prediction accuracies are generally superior to sequence-based tactics, which resulted in part from structure-based features that reflect DNA-binding or non-binding residues in a spatial point of view rather than sequence-based features. However, it must be satisfied that both the sequence of a given target protein and 3D structures are sufficiently available. Consequently, sequence-based computational techniques for the forecast about DNA-binding sites are more efficacious under practical conditions.

In this paper, a kind of sequence-based approach with ML is depicted. Conspicuously, PSSM information of protein sequence plays an important role in predicting DNA–protein binding sites according to the state-of-the-art investigations. Hence, we use PSSM information as the primary feature. The major difference between DNA-binding and non-DNA-binding proteins is that the functional binding sites are occurring in the former, whereas they are absent at the corresponding local regions of protein space in the latter. Moreover, protein functions incline to be evolutionarily conserved in these local regions. As a result, it requires that classifiers need to capture the hints of local functional conservation as fully as possible. Based on this leitmotiv, we devise an algorithm that is called Multi-scale Local Average Blocks (MLAB) to further extract local information from PSSM. The PSA information is also utilized to ameliorate the accuracy of prediction. Due to the number of DNA-binding residues (minority class) being significantly lower than that of non-binding residues (majority class), sample rescaling as straightforward strategy is adopted to deal with the issue of imbalanced data classification. To further handle the imbalanced problem, we employ an Ensemble Classifier with Random Under-Sampling (EC-RUS). Individual predictors of ensemble classifiers are realized by means of Weighted Sparse Representation based Classifier (WSRC). To evaluate the performance of our method, it has been validated through PDNA-543, PDNA-41, PDNA-316, PDNA-335 and PDNA-52 datasets. Our approach achieves MCC of 0.392, 0.315, 0.439 and 0.245 on PDNA-543, PDNA-41, PDNA-316 and PDNA-52, respectively. Experiments show that our method achieves better results than other outstanding methods. Compared with existing implementations, MCC for our algorithm are increased by at least 0.053, 0.015 and 0.064 on PDNA-543, PDNA-41 and PDNA-316, respectively.

## 2. Materials and Methods

For the sake of delving DNA–protein binding residues with computational methods, one of the major challenges is to fully describe the salient points of knowledge about DNA–protein binding sites in an adequate and concise way. Prediction of DNA–protein binding residues could be regarded as a traditional binary classification problem from the view of machine learning. Therefore, how to effectively extract feature from protein sequences turns out to be the preoccupation. Since the binding residue is not isolated from each other, we have the convention that 11 vicinal amino acid residues as a window (w=11), where the window specifically indicates the target residue and 5 neighbors on either side of the target residue itself. In light of this definition, an idiosyncratic multi-dimensional coding vector can be listed seriatim, which derives from the two aforementioned attributes of evolutionary conservation and predicted relative solvent accessibility. With the above information, the ML scenario is applied to build a prediction model about identifying DNA–protein binding sites.

### 2.1. Feature Extraction via Position Specific Scoring Matrix

By referring to the the form of Position Specific Scoring Matrix (PSSM), evolutionary conservation of protein sequence could be abstracted and generated by the de facto tool PSI-BLAST [41] (BLAST+ [42] options: -num_iterations 3 -db nr -inclusion_ethresh 0.001). The evolutionary information from PSSM is stored in a matrix of dimensions L×20 (*L* rows and 20 columns), formulated as

(1) $$PSSM={\left[\begin{array}{cccc} p_{1,1} & p_{1,2} & \cdots & p_{1,20}\\ p_{2,1} & p_{2,2} & \cdots & p_{2,20}\\ \vdots & \ddots & \vdots & \vdots \\ p_{L,1} & p_{L,2} & \cdots & p_{L,20} \end{array}\right]}_{L\times 20},$$

while each element in PSSM is calculated as

(2) $$\begin{array}{c} p_{i,j}=\sum_{k=1}^{20}\gamma \left(i,k\right)\times d\left(k,j\right)\;\left(i=1,\ldots ,L;j=1,\ldots ,20\right), \end{array}$$

where γ(i,k) is the frequency of *k*-th amino acid type at the position *i*; d(k,j) is the value of about the element in Dayhoff’s mutation matrix (substitution matrix), which corresponds to the amino acids between *k*-th and *j*-th type. The substitution matrix, which usually is wielded in DNA or protein sequence alignment, can describe the rate that certain kinds of characters in a protein sequence change to some other kind of character with time elapsing. As a supplementary, small values indicate that there is less conservatism in the corresponding areas, whereas large values indicate quite conservative zones.

These values are normalized to 0–1 range with min-max normalization. The original PSSM (see Equation(1)) is normalized as

(3) $$p_{i,j}^{\prime }=\frac{p_{i,j}-p_{min}}{p_{max}-p_{min}}\;\left(i=1,\ldots ,L;j=1,\ldots ,20\right),$$

where $p_{i,j}$ represents the original score of PSSM. While the normalized PSSM ($PSSM^{\prime }$) is represented as

(4) $$PSSM^{\prime }={\left[\begin{array}{cccc} p_{1,1}^{\prime } & p_{1,2}^{\prime } & \cdots & p_{1,20}^{\prime }\\ p_{2,1}^{\prime } & p_{2,2}^{\prime } & \cdots & p_{2,20}^{\prime }\\ \vdots & \ddots & \vdots & \vdots \\ p_{L,1}^{\prime } & p_{L,2}^{\prime } & \cdots & p_{L,20}^{\prime } \end{array}\right]}_{L\times 20}.$$

To distinguish DNA-binding and non-DNA-binding proteins, we need to justify the functional binding sites whether they occur at the corresponding local regions of protein space. As a further step of parenthetical explanation, protein functions in these local regions are inclined to be evolutionarily conserved. Considering this, we conceive an algorithm called Multi-scale Local Average Blocks (MLAB), which is enlightened by the Average Blocks (AB) approach that was proposed by Jeong et al. [43]. In virtue of extracting local information from normalized PSSM, Jeong et al. forecast protein function through ways that divide a protein sequence into *b* blocks and need not care much about the length of sequence. This idea has also been adopted in other bioinformatical issues [44]. On the occasion of this paper, each block consists of 20 features which are derived from 20 columns in PSSM. Similar to that, we formalize the value of attributes with a target by means of a window with 11 residues, and then obtain a vector of normalized PSSM scores whose gross amount is 11×20=220. However, it is quite imperative that, different from the AB algorithm, fixed size is changed into multi-scale size in our scheme; thus, the matrix is split in a horizontal manner. The PSSM-based Multi-scale Local Average Blocks (PSSM-MLAB) features can describe the relationship between target residue and neighboring residues in different resolutions.

**Figure 1:**
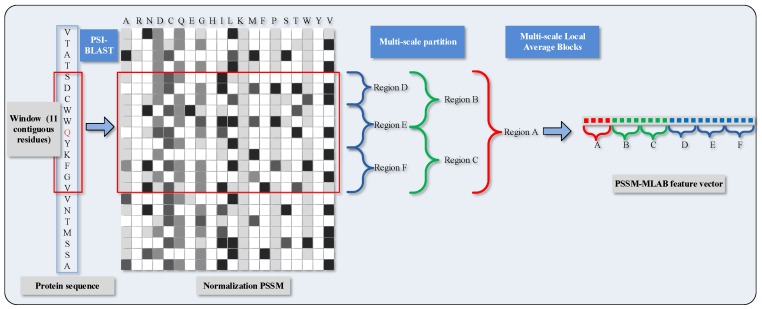
Schematic diagram of PSSM (Position Specific Scoring Matrix)-MLAB (Multi-scale Local Average Blocks) feature extraction.

More specifically, we partition the normalized PSSM of the target residues into six segmentations with varying composition, including global zone (A), bisection (B and C) and trichotomy (D, E and F). These segments can adequately portray multiple overlapping continuous and discontinuous interaction patterns that are schematically shown in Figure 1. The mean value of each local block is calculated with the formula as

(5) $$\begin{array}{c} LAB\left(k,j\right)=\frac{1}{B_{k}}\sum_{i=1}^{B_{k}}Mat_{k}\left(i,j\right)\;\left(i=1,\ldots ,B_{k};j=1,\ldots ,20;k=1,\ldots ,6\right), \end{array}$$

where LAB(k,j) regards the mean value of *k*-th block in the column *j*; $B_{k}$ stands for the amount of rows in block *k*; and $Mat_{k}\left(i,j\right)$ represents the value of cell in *i*-th row and *j*-th column of block *k*. Recapitulating the MLAB algorithm, through combining with normalized PSSM and partitioning manipulation on an entire sequence, we can gain a 6×20=120 dimensional feature vector.

### 2.2. Predicted Solvent Accessibility

Solvent accessibility has profound significance because it is closely affiliated with not only the spatial assignment of configuration, but also the swathing attitude about residues during the process of protein folding. It also coincides with the fact that there is a non-negligible association between solvent accessibility and DNA–protein interactions. The post hoc actuality has been instantiated, such as research by Ahmad et al. [45], who has demonstrated the importance of solvent accessibility to amino acid residues in predicting DNA–protein binding. By uniting the Solvent Accessibility prediction, which has been implemented with the de facto tool Nearest Neighbor method (SANN) [46], we can obtain the Predicted Solvent Accessibility (PSA) characteristics of each residue for the corresponding sequence. With min-max normalization, the PSA feature can also be normalized among a range from zero to one.

### 2.3. Weighted Sparse Representation Based Classifier

Sparse representation [47] as a sharp weapon of compressed sensing has aroused lots of scholarly pursuit for several years. Sparse representation-based classifier(SRC) [48,49] was firstly proposed by Wright et al. for the purpose about image recognition. In contrast to conventional taxonomization approaches such as SVM, KNN, RF, etc., SRC is robust for both outliers and noisy situations. To discriminate the sample corpus, which needs to be verified, SRC demands to create a Sparse Representation Matrix (SRM) and makes a linear combination on the training set. The reconstructed residuals of test sample for each kind of classification are measured and calculated through SRM and linear combination. Ultimately, the corpus of samples will be assigned to the corresponding category, arbitrated by minimum reconstruction residual. A group of researchers [50,51] have deployed SRC in solving issues in the area of computational biology.

Suppose there are totally *C* kind of classifications involved in a sufficient dataset. The assignment is how to correctly determine the attribution of a newly added sample y when we put it into the original corpus. SRC picks $n_{c}$ training samples from the *c*-th classification, which correspond to the volume of each raw in $x^{c}$. $x^{c}$ can be expressed as

(6) $$\begin{array}{c} x^{c}={\left[x_{1}^{c},\ldots ,x_{n_{c}}^{c}\right]}^{T}\;\left(1\leq c\leq C,x^{c}\in R^{n_{c}\times m^{\prime }}\right), \end{array}$$

where $m^{\prime }$ is the aggregate feature volume with regard to one sample. Thus, the training sample matrix can be written as

(7) $$\begin{array}{c} X={\left[x^{1},\ldots ,x^{c},\ldots ,x^{C}\right]}^{T}\;\left(X\in R^{n\times m^{\prime }}\right), \end{array}$$

where $n=\sum_{c=1}^{C}n_{c}$ represents the amount of training samples. Then, the known test sample y will approximately fall in the linear spanning region of the training samples associated with the *c*-th classification as

(8) $$y^{c}=\alpha_{0}^{c}x^{c}.$$

While under unknown *c* condition, test sample y will be in line with the representation of whole training set using linear regression as

(9) $$y=\alpha_{0}X\;,$$

where coefficient vector is $\alpha_{0}=\left[0,\cdots ,\alpha_{0}^{c},\cdots ,0\right]$; vector $\alpha_{0}^{c}$ which is associated with the *c*-th classification is non-zero.

$\alpha_{0}$ could take on a kind of sparse state, whereas the size of sample about corresponding classification is huge. The critical step of SRC algorithm is selecting the α vector that can both satisfy Equation (9) and minimize the $l_{0}$-norm per se with the equations as

(10) $$\begin{array}{c} \hat{\alpha_{0}}=\mathrm{argmin}{∥\alpha ∥}_{0},\\ s.t.\;y=\alpha X. \end{array}$$

Unfortunately, searching the sparsest solution for equations (10) is NP (Non-deterministic Polynomial)-hard. Still, as a remedy, by means of solving $l_{1}$-minimization problem which belongs to convex optimization, we can eschew the $l_{0}$-minimization problem since $l_{1}$-minimization problem can be viewed as problem that is approximately equivalent to $l_{0}$-minimization. For the sake of resolving this occasion in $l_{1}$, Equation (10) can be transformed into an expression as

(11) $$\begin{array}{c} \hat{\alpha_{1}}=\mathrm{argmin}{∥\alpha ∥}_{1},\\ s.t.\;\hat{y}=\alpha X,\\ \;∥y-\hat{y}∥\leq \epsilon \;\left(\epsilon >0\right), \end{array}$$

where ϵ reflects the tolerance of reconstruction deviation.

The SRC approach allocates the label of test pattern *y* w.r.t. category *c* according to equations

(12) $$\left\{\begin{array}{cc} v_{y} & =\min v_{y}^{c},\\ v_{y}^{c} & =∥y-\hat{\alpha_{1}^{c}}{X∥}_{2}, \end{array}\right.$$

where $v_{y}^{c}$ denotes the residuals between *y* and $\hat{\alpha_{1}^{c}}X$ (category *c*). Thus, $v_{y}=\min v_{y}^{c}$ means sample *y* will be assigned to the category that owns minimal residuals.

To fix the problem about instability of SRC which may be aroused by noise pollution, Lu et al. [52] have proposed the Weighted Sparse Representation based Classification (WSRC) method, which deals with the whole training set as a vocabulary, and imposes the locality on the weighted $l_{1}$ regularization.

(13) $$\begin{array}{c} \hat{\alpha_{1}}=\mathrm{argmin}{∥\alpha \Lambda ∥}_{1},\\ s.t.\;∥y-\alpha X∥\leq \epsilon , \end{array}$$

where Λ is a diagonal matrix about locality adaptor as

(14) $$\Lambda ={\left[\begin{array}{cccc} \lambda_{1,1} & 0 & \cdots & 0\\ 0 & \lambda_{2,2} & \cdots & 0\\ \vdots & \ddots & \vdots & \vdots \\ 0 & 0 & \cdots & \lambda_{C,C} \end{array}\right]}_{C\times C}.$$

Moreover, λ denotes to the Euclidean distance from y to $x_{i}^{c}$ , which is expressed as

(15) $$\lambda =\exp(-\frac{∥y-x_{i}^{c}{∥}^{2}}{2\sigma^{2}}),$$

where *i* indicates the sample index of training set w.r.t. category *c*. σ corresponds to the Gaussian kernel width. y, $x_{i}^{c}$ represents test sample and training sample, respectively. In addition, the values of Gaussian distance can be viewed as the weight of each sample in training sets.

Nevertheless, the output of WSRC just meets a straightforward preliminary mapping that each residual corresponds to a certain kind of classification (without prediction score w.r.t. each category), since there is a greater possibility for the minimum residue of the corresponding category. For the convenience of projecting the output about WSRC between [0,1], there are three types of scores for binding sites prediction, which are represented as

(16) $$score_{1}\left(y\right)=2^{-v_{binding}\left(y\right)/v_{non-binding}\left(y\right)}\;,$$

(17) $$score_{2}\left(y\right)=1-\frac{v_{binding}\left(y\right)}{v_{non-binding}\left(y\right)+v_{binding}\left(y\right)}\;,$$

(18) $$score_{3}\left(y\right)=\frac{1}{1+e^{-(v_{non-binding}\left(y\right)-v_{binding}\left(y\right))}}\;,$$

where $v_{binding}\left(y\right)$ and $v_{non-binding}\left(y\right)$ are the deviation of reconstruction about WSRC when assigning test sample y w.r.t. binding and non-binding site, respectively. The assessment about the performance with the aforementioned three types of score is shown in the experimental evaluation section. In practice from our research, $v_{1}\left(y\right)=v_{binding}\left(y\right)$ and $v_{2}\left(y\right)=v_{non-binding}\left(y\right)$.

The accomplishment of feature extraction implies that no matter the target residue binding sites or non-binding sites, all of them have been converted to numerical feature vectors that have their own identical dimension. The feature space for every target residue binding site is comprised of two parts that are PSSM-MLAB features ($f_{PSSM-MLAB}$) and PSA features ($f_{PSA}$), respectively. It needs to be reaffirmed that all of the feature vectors have been normalized by means of min-max normalization.

### 2.4. Ensemble Classifier and Random Under-Sampling

Imbalanced datasets, which are characterized as a larger ratio size between non-binding examples (majority category) and binding examples (minority category), always exist in the issue of classification. Exploiting a schema with ensemble classifier [53,54] is a fashionable way. Consequently, we exploit an ensemble of *m* classifiers with bootstrap resampling strategy [53,54]. By performing random sampling on *m* subsets, which also be considered with replacement, from the majority category of non-binding examples, we can make all negative subsets own the same or similar size as the minority category of binding examples. After this step, every negative subset will group with the set of binding cases and generate *m* new training sets. Thus, *m* classifiers, which are represented as ${\left\{f{\left(x\right)}_{i}\right\}}_{i=1}^{m}$, can be built according to the *m* training sets. Finally, the outcome is voted by arithmetic mean value of the results that come from *m* sub-classifiers. After calculating about every score as $score{\left(y\right)}^{i}$, we can get the final rate of voting P(y) by

(19) $$P\left(y\right)=\frac{1}{m}\sum_{i=1}^{m}score{\left(y\right)}^{i}\;,$$

where P(y) also denotes the probabilistic factor of test sample *y*, and $score{\left(y\right)}^{i}$ reflects the probability value of *i*-th base classifier.

**Figure 2:**
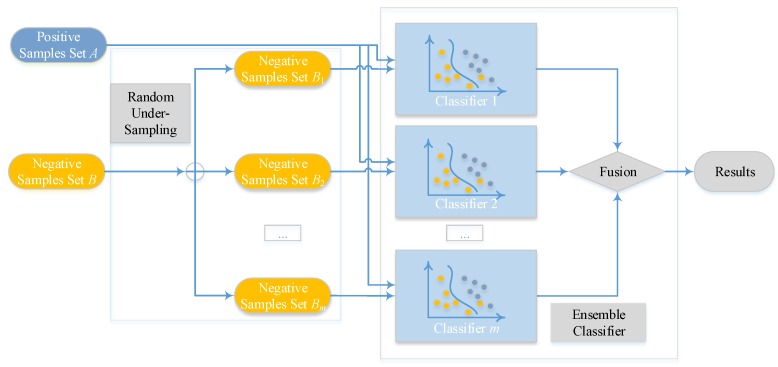
Overview of the ensemble classifier.

The overview of the proposed ensemble model is shown in Figure 2, Ensemble Classifier with Random Under-Sampling (EC-RUS) as the scenario to deal with the imbalanced issue.

## 3. Results

We test our method on several DNA–protein binding sites datasets to evaluate the performance of our proposed approach, including PDNA-543, PDNA-41 (independent test set of PDNA-543), PDNA-335, PDNA-52 (independent test set of PDNA-335) and PDNA-316. First, we independently analyze the performance of binding site representations, such as PSSM, PSSM-MLAB and PSA. Second, we compare our method with some outstanding methods on the above datasets.

### 3.1. Datasets of DNA–Protein Binding Sites

PDNA-543 and PDNA-41 are independent test datasets that have been constructed by Hu et al. [13]. They collect a dataset that contains 7,186 DNA-binding protein chains and has clear target annotations in PDB (Protein Data Bank) [55]. After removing redundant sequences by wielding CD-hit software [56], there are totally 584 non-redundant protein sequences that can be obtained and no two sequences had more than 30% identity. Then, they divide the non-redundant sequences into two sections, which are the training dataset (PDNA-543) and the independent test dataset (PDNA-41).

PDNA-335 and PDNA-52 are independent test datasets that have been employed by Yu et al. [57]. In their research, all of the protein sequences are extracted, which are based on BioLip [58] rather than on PDB [55]. Next, the maximal pairwise sequential identity of the extracted protein sequences are culled to a 40 percent level by using PISCES software (1.0, Wang, G. and Roland, L. Dunbrack Jr, Philadelphia, PA, USA) [59]. The remaining sequences constitute the training dataset. Besides that, the test set is extracted in a similar process. Moreover, if a given sequence in the validation dataset shares more than 40 percent similarity to a sequence in the training dataset, then remove the sequence from the validation dataset. Training set and independent validation test sets contain 335 and 52 protein sequences, respectively.

PDNA-316 is constructed by Si et al. [12]. The dataset embraces 316 DNA-binding protein chains, 5609 binding sites and 67,109 non-binding sites. The detailed information of PDNA-543, PDNA-41, PDNA-335, PDNA-52 and PDNA-316 is summarized in Table 1. Related datasets, codes, and figures of our algorithm are available https://github.com/6gbluewind/PRODNA.

### 3.2. Evaluation Measurements

To test the robustness, the process of random selection about training and test sets, model-building and model-evaluating are performed repeatedly, which deals with the manner of ten-fold cross validation. Seven parameters, including overall prediction accuracy (ACC), sensitivity (SN), specificity (Spec), positive predictive value (Pre), and Matthew’s correlation coefficient (MCC) are used in the assessing procedure. These parameters are represented as

(20) $$ACC=\frac{TP+TN}{TP+FP+TN+FN}\;,$$

(21) $$SN=\frac{TP}{TP+FN}\;,$$

(22) $$Spec=\frac{TN}{TN+FP}\;,$$

(23) $$Pre=\frac{TP}{TP+FP}\;,$$

(24) $$MCC=\frac{TP\times TN-FP\times FN}{\sqrt{\left(TP+FN)\times (TN+FP)\times (TP+FP)\times (TN+FN\right)}}\;,$$

where true positive (TP) is the number of true DNA–protein binding sites that are predicted correctly; false negative (FN) is the number of true DNA–protein binding sites that are predicted to be non-binding; false positive (FP) is the number of true non-binding sites that are predicted to be binding sites, and true negative (TN) is the number of true non-binding sites that are predicted correctly.

The Area Under the Receiver Operating Characteristic (AUC) is a common summary statistic that can measure the goodness of a predictor in a binary classification task. It is equal to the probability that a predictor will rank a randomly chosen positive instance higher than a randomly chosen negative one. We would like to emphasize that two WSRC parameters are set to σ=1.5 and ϵ=0.5, respectively.

### 3.3. Predicted Results on the PDNA-543 Dataset

#### 3.3.1. Selecting Optimal Size of Sliding Window and Number of Base Classifiers

Different sizes of sliding window may lead to different performance. In addition, the number of base classifiers will also affect the appearance of prediction. In Hu’s work [13], they use two strategies for selecting Thresholds (*T*): (1) they selected the threshold that makes Sen≈Spec, and (2) they select the threshold that makes FPR≈5% ( FPR=1-Spec ). In virtue of that, we adjust window size from 7 to 17 residues and number of base classifiers (*m*) from 1 to 29, with a step size of 2, on PDNA-543 dataset over a ten-fold cross-validation with above two strategies. Hitherto, we select Equation (16) as default score function of base classifiers (WSRC, Equation (16)).

**Figure 3:**
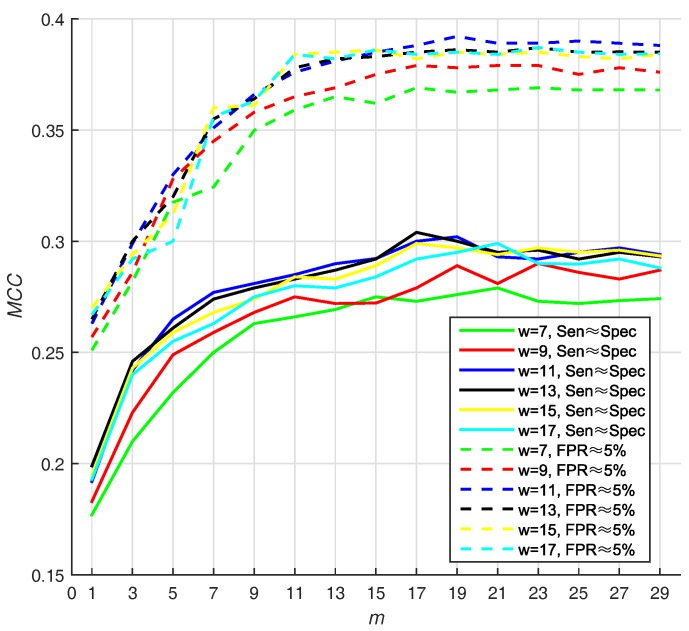
The MCC (Matthew Correlation Coefficient) of PSSM (Position Specific Scoring Matrix)- MLAB (Multi-scale Local Average Blocks) with different sizes of sliding window and numbers of base classifiers (WSRC (Weighted Sparse Representation based Classifier), Equation(16)).

We select the optimal size by highest MCC value, and find that 11 and 19 are the best parameters of window size, whereas number of base classifiers under FPR≈5%. Under the condition that Sen≈Spec, the value of MCC is also high (*w* = 11, *m* = 19). The result w.r.t. PDNA-543 is shown in Figure 3. As seen from the dotted curves, the MCC increases when size increases from 7 to 11. However, it slightly declines when size increases from 11 up to 17. The first maximum MCC value is achieved when m=19 (under FPR≈5%), and no improvement can be observed with larger values of *m*. Recapitulating these results, we set the optimal *m* as 19 in the investigation. The best MCC is 0.392, when window size and number of base classifiers are 11 residues and 19 with under FPR≈5%, respectively.

#### 3.3.2. Performance of Different Features

**Figure 4:**
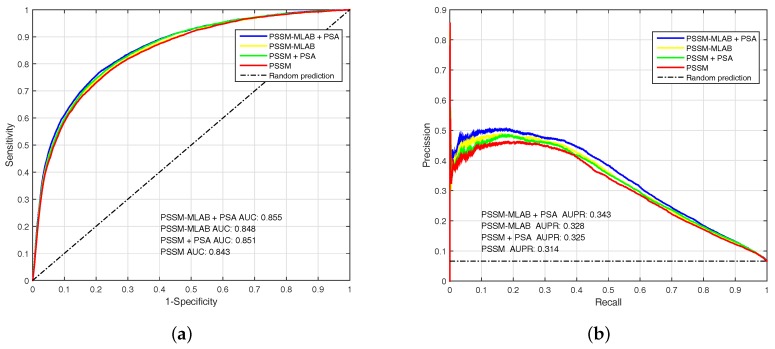
The AUC (Area Under the Receiver Operating Characteristic) and AUPR (Area Under the Precision-Recall curve) of PSSM (Position Specific Scoring Matrix), PSSM + PSA (Predicted Solvent Accessibility), PSSM-MLAB (Multi-scale Local Average Blocks) and PSSM-MLAB + PSA obtained with EC-RUS (Ensemble Classifier with Random Under-Sampling) (WSRC (Weighted Sparse Representation based Classifier), Equation (16)) on PDNA (Protein and DNA)-543 dataset over a ten-fold cross-validation test. (**a**) receiver operating characteristic curves; (**b**) precision–recall curves.

To analyze the performance of PSSM, PSSM-MLAB and PSA features, we evaluate these features by EC-RUS on PDNA-543 dateset. Results for PSSM, PSSM + PSA, PSSM-MLAB and PSSM-MLAB + PSA are shown in Table 2 and Figure 4. In addition, Equation (16) is also selected as default score function of base classifiers (WSRC, Equation (16)). The MCC (under FPR≈5%) of PSSM, PSSM + PSA, PSSM-MLAB and PSSM-MLAB + PSA are 0.364, 0.375, 0.378 and 0.392, respectively. Obviously, the combinatorial approach of PSSM-MLAB + PSA achieves better performance than PSSM, PSSM + PSA or PSSM-MLAB. Furthermore, the MCC of PSSM-MLAB (0.378) is higher than PSSM (0.364) and PSSM + PSA (0.375). Consequently, the MLAB algorithm can reduce the dimension of PSSM and remove some noise. Because of additional solvent accessibility information, the MCC (under FPR≈5%) of PSSM-MLAB + PSA (0.392) and PSSM + PSA (0.375) are all higher than single PSSM-MLAB (0.378) and single PSSM (0.364), respectively. In Figure 4, we can see that the fusion feature of PSSM-MLAB and PSA has better performance than the other features in the PDNA-543 dataset.

#### 3.3.3. Selecting Optimal Score Function of Base WSRC

**Figure 5:**
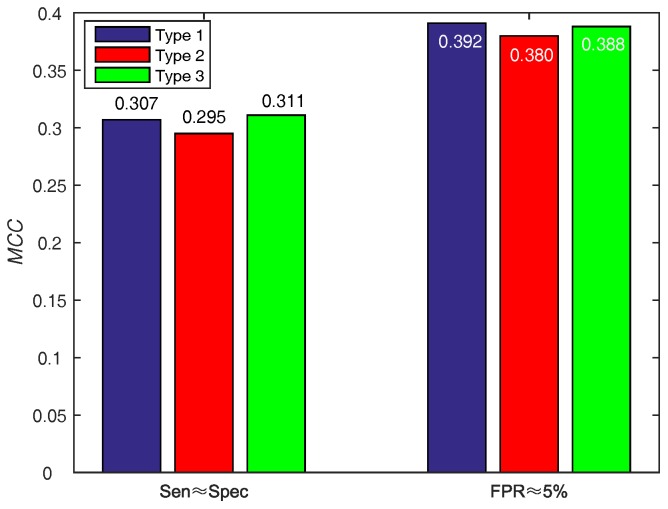
Results for different score functions on PDNA-543. Type 1, 2 and 3 represent Equations (16)–(18), respectively.

In order to make a decision w.r.t. score function (WSRC) from Equations (16)–(18), we evaluate the above functions on PDNA-543 across a ten-fold cross-validation test with PSSM-MLAB + PSA as feature. The results of different functions on PDNA-543 are shown in Figure 5. Obviously, the performance of three different score functions are almost identical. However, the type 1 (0.392, under FPR≈5%) function achieves better performance of MCC than type 2 (0.380, under FPR≈5%) and 3 (0.388, under FPR≈5%). Thus, we select the Equation (16) as the score function of WSRC.

#### 3.3.4. Comparison with Existing Predictors on PDNA-543

We also compare the prediction performance of our proposed method with Hu’s work [13] on this dataset, as shown in Table 3. Our method achieves 0.307 MCC under Sen≈Spec. However, Hu’s work achieves 0.304 MCC under Sen≈Spec. Moreover, our method achieves the best MCC of 0.392 under FPR≈5%. Our method obtains better prediction results than Hu’s work on the PDNA-543 dataset.

### 3.4. Predicted Results on the Independent Test Set of PDNA-41

**Figure 6:**
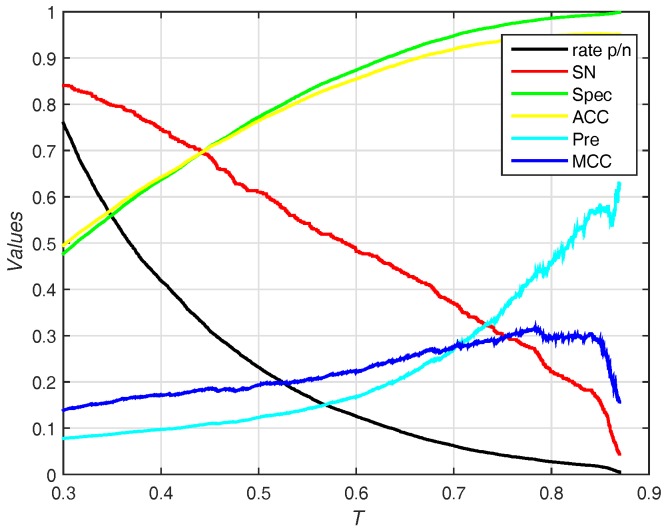
Results for different thresholds of probability on independent test set of PDNA-41. Rate p/n means the ratio between the predictive number of binding sites and the predictive number of non-binding sites.

In this section, we use the PDNA-543 dataset as the training set and PDNA-41 as the independent test set. It has been compared with other previous works including BindN [3], ProteDNA [9], BindN+ [5], MetaDBSite [12], DP-Bind [60], DNABind [20] and TargetDNA [13] with summarizing results in Table 4. Under FPR≈5%, our method (EC-RUS built with WSRC) achieves 0.9458 accuracy, 0.2725 sensitivity, 0.4292 Pre and 0.315 MCC. Comparing with Sen≈Spec, sensitivity declines (0.3379), specificity, accuracy, Pre and MCC rise together (0.2006, 0.1814, 0.3061 and 0.122, respectively). Furthermore, our method achieves the best MCC (0.315) under FPR≈5%. Figure 6 shows the trend (including sensitivity, specificity, accuracy, Pre and MCC) on different threshold *T* of probability. While the threshold of probability rises, values of specificity, accuracy, Pre and MCC are synchronously rising. The trend of sensitivity and rate p/n are opposite.

In addition, we test different types of base classifiers to build an EC-RUS model. The base classifiers together contain SVM [25,61], RF [26], L1-regularized Logistic Regression (L1-LR) [62] and Sparse Bayesian Learning (SBL) [63]. Under FPR≈5%, EC-RUS (SVM), EC-RUS (RF), EC-RUS (L1-LR) and EC-RUS (SBL) achieve MCC of 0.302, 0.261, 0.246 and 0.247, respectively. The MCC (0.315) EC-RUS (WSRC) is better than the above models. We can see that a sparse representation based classifier is suitable for the classification with PSSM-MLAB features. The best performance lies in the fact that weighted SRC further improves the performance of basic SRC and the easily adjusted parameter of WSRC can exert its effect fully in our experiments.

### 3.5. Predicted Results on the PDNA-316 Dataset

In order to highlight the advantage of our method, we also test on the PDNA-316 dataset, which is described by Si et al. [12]. We compare the prediction performance of our proposed method with other previous works including DBS-PRED [7], BindN [3], DNABindR [6], DISIS [11], DP-Bind [60], BindN-RF [4], MetaDBSite [12] and TargetDNA [13]. In Table 5, we can see that the average prediction performance of our method, such as sensitivity, specificity, accuracy and MCC are 0.8067, 0.7818, 0.7837 and 0.356 under Sen≈Spec, respectively. Although DISIS [11] achieves better values of specificity and ACC, and ACC, which are 0.9800, 0.9200, the sensitivity (0.1900) and MCC (0.250) are not high. Furthermore, our method (EC-RUS built with WSRC) achieves the best MCC of 0.439 under FPR≈5%. It is shown that the MLAB algorithm deeply extracts the evolutional information from PSSM.

Different types of base classifiers are used to construct the EC-RUS model, and EC-RUS (SVM), EC-RUS (RF), EC-RUS (L1-LR) and EC-RUS (SBL) achieve MCC (under FPR≈5%) of 0.426, 0.394, 0.319 and 0.317, respectively. Extensive experiments in our study have illustrated that WSRC is better than other classifiers.

### 3.6. Predicted Results on PDNA-335 and PDNA-52 Datasets

PDNA-335 and PDNA-52 (independent test set of PDNA-335) are collected by Yu et al. [57]. To further evaluate our model, we employ the PDNA-335 dataset as the training set and PDNA-52 as the independent test set. Performance comparison about our method with TargetS [57], MetaDBSite [12], DNABR [64], and alignment-based predictor on the independent validation dataset of PDNA-52 is listed in Table 6. At the occasion of imbalanced learning, the MCC provides the overall measurement about the quality of binary prediction. In Yu’s work [57], they implement the evaluation by choosing the Threshold (T) of probability value, in order to maximize the MCC value of prediction. Thus, we apply the same evaluation on PDNA-52. Obviously, TargetS achieves the best overall prediction performance among the nine listed predictors with the highest MCC value of 0.377, which is about 0.13 higher than that of the second-best (0.245) performer model (EC-RUS built with WSRC). The proposed method along with TargetS does not perform well on an independent PDNA-52 dataset because TargetS has equipped the residues’ 3D coordinates contained in the PDB file to spatial clustering before it probes binding sites. Results of experiments show that our model is also compatible by comparing with the rest of the methods on an independent PDNA-52 dataset.

**Figure 7:**
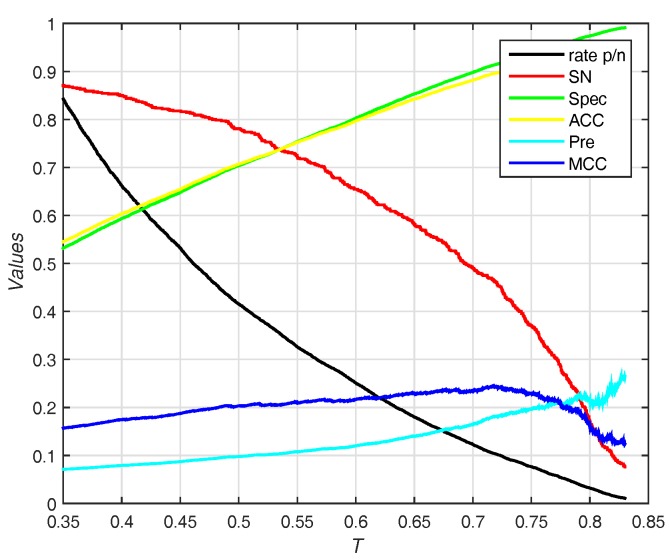
Results for different thresholds of probability on Independent test set of PDNA-52. Rate p/n means the ratio between the predictive number of binding sites and the predictive number of non-binding sites.

The trends (including sensitivity, specificity, accuracy, Pre and MCC) on different threshold *T* of probability are shown in Figure 7. EC-RUS (WSRC) achieves the highest MCC of 0.245 when T=0.718.

### 3.7. Significance Analysis

We employ the Wilcoxon rank-sum test to analyze the statistical significance of MCC between other methods (including MetaDBSite and TargetDNA) and our method on PDNA-543, PDNA-41, PDNA-316 and PDNA-52 datasets. The significance level is 0.05, and results of tests are shown in Table 7. The differences between other methods and our method are not significant (MetaDBSite *p*-value : 0.2667, TargetDNA *p*-value : 0.4610). The main reason of this is that most of the above methods are based on sequence information. Hence, the increment of MCC is small. We would consider structure information in our further work.

### 3.8. Case of Prediction

**Figure 8:**
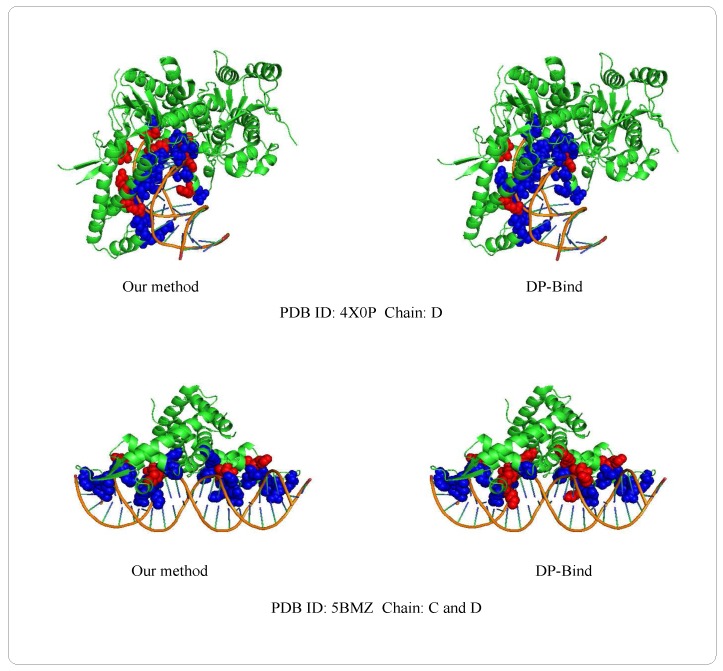
Representative protein-DNA complex: Upper is 4X0P-D (PDB ID: 4X0P, Chain: D), lower is 5BMZ-CD (PDB ID: 5BMZ, Chain: C and D).

Examples of 4X0PD (PDB ID: 4X0P, Chain: D) and 5BMZCD (PDB ID: 5BMZ, Chain: C and D) belong to the PDNA-41 dataset. We use the PDNA-543 dataset as the training set to predict two examples, which are shown in Figure 8. The orange object of the helix is a DNA chain, while the green object is the protein sequence (containing helix, fold and loop structure). Blue regions and red regions are the true prediction and false prediction, respectively. In addition, the results of two methods (our method and DP-Bind [60]) are shown in Table 8. On the 4X0P-D, the FP of our method (34) is less than DP-Bind (154). Furthermore, the FP and FN of our method (9, 3) are both less than DP-Bind (16, 7) on 5BMZ-D.

### 3.9. Running Time

The computational complexity and running time of WSRC depend on the number of training and testing samples. The Gaussian distances of testing and each training sample are calculated by the WSRC. The running time of other classifiers only depend on the number of training sets. The sizes of PDNA-543 (Training sets) and PDNA-41 (Testing sets) are 144,544 and 14,755, respectively. The PDNA-335 (Training sets) and PDNA-52 (Test sets) contain 77,781 and 17,198 samples, respectively. Although the WSRC is time-consuming, the performance is better than other classifiers on PDNA-41 and PDNA-52. The running times are listed in Table 9.

## 4. Discussion

Albeit many computational approaches have been proposed to prospect DNA–protein binding sites, there still have potential enhancing space for refining the state-of-the-art prediction models. Existing methods always disregard local environments that appear neither reliable nor robust. Hence, we put forward a kind of multi-scale local average blocks idea to further leach local evolutionary information from PSSM. Compared with original PSSM, the MCC of PSSM-MLAB rises by 0.014 in a PDNA-543 dataset.

## 5. Conclusions

Our algorithm has been extensively validated on several datasets including PDNA-543, PDNA-41, PDNA-316, PDNA-335 and PDNA-52 datasets. Our method achieves MCC of 0.392, 0.315, 0.439 and 0.245 on PDNA-543, PDNA-41, PDNA-316 and PDNA-52 datasets, respectively. Contrasted with the state-of-the-art prediction models, MCC (under FPR≈5%) for our method is increased by at least 0.053, 0.015 and 0.064 on PDNA-543, PDNA-41 and PDNA-316 datasets, respectively. Our method has reached a desired performance that achieves comparable or even better prediction results across possessive datasets. Besides that, our method could be a de facto instrument for future proteomics studies.

In the future, we will ameliorate the forecasting performance of MLAB by refining the feature representation and classification tactics. For feature representation, we will consider the amino acid compositions and predicted secondary structures, which have been obtained as local PSSM-based features. Furthermore, well-established classifier also can be an alternative condition. Powerful classifiers such as Modified AdaBoost(MAdaBoost) [57] and LibD3C [65,66] can all be integrated into the clustering and dynamic selecting schema.

## Figures and Tables

**Table 1:** Four different datasets of DNA–protein binding sites.

Dataset	No. of Sequences	No. of Binding ${}^{a}$	No. of Non-Binding ${}^{b}$	Ratio ${}^{c}$
PDNA(Protein and DNA)-543	543	9549	134,995	14.137
PDNA-41	41	734	14,021	19.102
PDNA-335	335	6461	71,320	11.038
PDNA-52	52	973	16,225	16.675
PDNA-316	316	5609	67,109	11.964

${}^{a}$: No. of Binding represents the number of positive samples. ${}^{b}$: No. of Non-Binding represents the number of negative samples. ${}^{c}$: Ratio = No. of Non-Binding / No. of Binding.

**Table 2:** The performance comparison of different features through ten-fold cross-validation by EC-RUS (Ensemble Classifier with Random Under-Sampling) (WSRC (Weighted Sparse Representation based Classifier), Equation (16)) on PDNA-543 dataset.

Feature	SN	Spec	ACC	Pre	MCC	AUC
PSSM (Sen≈Spec)	0.7738	0.7570	0.7581	0.1844	0.294	0.843
PSSM (FPR≈5%)	0.4377	0.9500	0.9160	0.3832	0.364	0.843
PSSM + PSA (Sen≈Spec)	0.7850	0.7590	0.7607	0.1874	0.302	0.851
PSSM + PSA (FPR≈5%)	0.4541	0.9494	0.9166	0.3886	0.375	0.851
PSSM-MLAB (Sen≈Spec)	0.7744	0.7599	0.7609	0.1864	0.297	0.848
PSSM-MLAB (FPR≈5%)	0.4516	**0.9510**	0.9178	0.3955	0.378	0.848
PSSM-MLAB + PSA (Sen≈Spec)	**0.7894**	0.7629	0.7646	0.1907	0.307	**0.855**
PSSM-MLAB + PSA (FPR≈5%)	0.4762	0.9492	**0.9180**	**0.3991**	**0.392**	**0.855**

**Table 3:** Comparison with the TargetDNA on PDNA-543 dataset by ten-fold cross-validation.

Methods	SN	Spec	ACC	Pre	MCC	AUC
TargetDNA (Sen≈Spec) ${}^{*}$ [13]	0.7698	0.7705	0.7704	0.1918	0.304	0.845
TargetDNA (FPR≈5%) ${}^{*}$ [13]	0.4060	**0.9500**	0.9140	0.3647	0.339	0.845
Our method (Sen≈Spec)	**0.7894**	0.7629	0.7646	0.1907	0.307	**0.855**
Our method (FPR≈5%)	0.4762	0.9492	**0.9180**	**0.3991**	**0.392**	**0.855**

Results excerpted from [13].

**Table 4:** Comparison with some state-of-the-art works on the Independent PDNA-41 dataset.

Methods	MCC	SN	Spec	ACC	Pre
BindN ${}^{*}$	0.143	0.4564	0.8090	0.7915	0.1112
ProteDNA ${}^{*}$	0.160	0.0477	**0.9984**	**0.9511**	**0.6030**
BindN+ (FPR≈5%) ${}^{*}$	0.178	0.2411	0.9511	0.9158	0.2051
BindN+ (Spec≈85%) ${}^{*}$	0.213	0.5081	0.8541	0.8369	0.1542
MetaDBSite ${}^{*}$	0.221	0.3420	0.9335	0.9041	0.2122
DP-Bind ${}^{*}$	0.241	0.6172	0.8243	0.8140	0.1553
DNABind ${}^{*}$ (structure based)	0.264	0.7016	0.8028	0.7978	0.1570
TargetDNA (Sen≈Spec) ${}^{*}$	0.269	0.6022	0.8579	0.8452	0.1816
TargetDNA (FPR≈5%) ${}^{*}$	0.300	0.4550	0.9327	0.9089	0.2613
EC-RUS (WSRC) (Sen≈Spec) ${}^{a}$	0.193	0.6104	0.7725	0.7644	0.1231
EC-RUS (WSRC) (FPR≈5%) ${}^{a}$	**0.315**	0.2725	0.9731	0.9458	0.4292
EC-RUS (SVM) (Sen≈Spec) ${}^{a}$	0.261	0.6975	0.8032	0.7972	0.1567
EC-RUS (SVM) (FPR≈5%) ${}^{a}$	0.302	0.3787	0.9577	0.9281	0.3092
EC-RUS (RF) (Sen≈Spec) ${}^{a}$	0.234	0.6785	0.7818	0.7767	0.1401
EC-RUS (RF) (FPR≈5%) ${}^{a}$	0.261	0.3351	0.9524	0.9217	0.2691
EC-RUS (L1-LR) (Sen≈Spec) ${}^{a}$	0.228	0.6199	0.8084	0.7991	0.1449
EC-RUS (L1-LR) (FPR≈5%) ${}^{a}$	0.246	0.3120	0.9541	0.9221	0.2623
EC-RUS (SBL) (Sen≈Spec) ${}^{a}$	0.219	**0.7084**	0.7434	0.7416	0.1263
EC-RUS (SBL) (FPR≈5%) ${}^{a}$	0.247	0.3202	0.9521	0.9206	0.2591

*: Results excerpted from [13]. ${}^{a}$: The feature is PSSM-MLAB + PSA. In addition, the EC-RUS model is built with different base classifiers.

**Table 5:** Comparison of the prediction performance between the proposed method and some state-of-the-art works on PDNA-316 dataset.

Methods	SN	Spec	ACC	MCC
DBS-PRED ${}^{*}$ (structure based)	0.5300	0.7600	0.7500	0.170
BindN ${}^{*}$	0.5400	0.8000	0.7800	0.210
DNABindR ${}^{*}$	0.6600	0.7400	0.7300	0.230
DISIS ${}^{*}$	0.1900	**0.9800**	**0.9200**	0.250
DP-Bind ${}^{*}$	0.6900	0.7900	0.7800	0.290
BindN-RF ${}^{*}$	0.6700	0.8300	0.8200	0.320
MetaDBSite [12]	0.7700	0.7700	0.7700	0.320
TargetDNA (Sen≈Spec) [13]	0.7796	0.7803	0.7802	0.339
TargetDNA (FPR≈5%) [13]	0.4302	0.9500	0.9099	0.375
EC-RUS (WSRC) (Sen≈Spec) ${}^{a}$	**0.8067**	0.7818	0.7837	0.356
EC-RUS (WSRC) (FPR≈5%) ${}^{a}$	0.5108	0.9499	0.9161	**0.439**
EC-RUS (SVM) (Sen≈Spec) ${}^{a}$	0.8011	0.7969	0.7973	0.369
EC-RUS (SVM) (FPR≈5%) ${}^{a}$	0.4935	0.9500	0.9150	0.426
EC-RUS (RF) (Sen≈Spec) ${}^{a}$	0.7989	0.7542	0.7576	0.326
EC-RUS (RF) (FPR≈5%) ${}^{a}$	0.4521	0.9502	0.9118	0.394
EC-RUS (L1-LR) (Sen≈Spec) ${}^{a}$	0.7347	0.7659	0.7635	0.300
EC-RUS (L1-LR) (FPR≈5%) ${}^{a}$	0.3523	0.9498	0.9037	0.319
EC-RUS (SBL) (Sen≈Spec) ${}^{a}$	0.7453	0.7540	0.7533	0.295
EC-RUS (SBL) (FPR≈5%) ${}^{a}$	0.3562	0.9480	0.9023	0.317

*: Results excerpted from [12,13]. ${}^{a}$: The feature is PSSM-MLAB + PSA. In addition, EC-RUS model is built with different base classifiers.

**Table 6:** Comparison with some state-of-the-art works on PDNA-52 dataset under maximizing the value of MCC.

Methods	SN	Spec	ACC	MCC	AUC
TargetS [57] ${}^{*}$	0.413	**0.965**	**0.933**	**0.377**	**0.836**
MetaDBSite [12] ${}^{*}$	0.580	0.764	0.752	0.192	-
DNABR [64] ${}^{*}$	0.407	0.873	0.846	0.185	-
alignment-based ${}^{*}$	0.266	0.943	0.905	0.190	-
EC-RUS (WSRC) ${}^{a}$	0.467	0.913	0.896	0.245	0.808
EC-RUS (SVM) ${}^{a}$	0.528	0.835	0.823	0.185	0.756
EC-RUS (RF) ${}^{a}$	0.561	0.773	0.764	0.152	0.741
EC-RUS (L1-LR) ${}^{a}$	0.594	0.811	0.803	0.201	0.787
EC-RUS (SBL) ${}^{a}$	**0.635**	0.782	0.776	0.192	0.786

*: Results excerpted from [57]. ${}^{a}$: The feature is PSSM-MLAB + PSA. In addition, EC-RUS model is built with different base classifiers.

**Table 7:** The statistical significance of MCC between other methods (including MetaDBSite and TargetDNA) and our method.

Methods	*p*-Value
Our method-MetaDBSite	0.2667
Our method-TargetDNA	0.4610

**Table 8:** Comparison with DP (DNA Protein)-Bind on 4X0P-D and 5BMZ-D.

PDB (Protein Data Bank) ID	Method	TP	TN	FP	FN
4X0P-D	our method	24	559	34	8
	DP-Bind	29	439	154	3
5BMZ-D	our method	14	110	9	3
	DP-Bind	10	103	16	7

**Table 9:** The running time (seconds) of EC-RUS on PDNA-41 and PDNA-52 independent testing sets.

Classifier	PDNA-41	PDNA-52
EC-RUS (WSRC)	9227	14,407
EC-RUS (L1-LR)	705	232
EC-RUS (RF)	3778	1632
EC-RUS (SBL)	136,241	40,121
EC-RUS (SVM)	27,210	2043
